# Gust Mitigation of Micro Air Vehicles Using Passive Articulated Wings

**DOI:** 10.1155/2014/598523

**Published:** 2014-01-02

**Authors:** Adetunji Oduyela, Nathan Slegers

**Affiliations:** School of Mechanical and Aerospace Engineering, University of Alabama in Huntsville, Huntsville, AL 35899, USA

## Abstract

Birds and insects naturally use passive flexing of their wings to augment their stability in uncertain aerodynamic environments. In a similar manner, micro air vehicle designers have been investigating using wing articulation to take advantage of this phenomenon. The result is a class of articulated micro air vehicles where artificial passive joints are designed into the lifting surfaces. In order to analyze how passive articulation affects performance of micro air vehicles in gusty environments, an efficient 8 degree-of-freedom model is developed. Experimental validation of the proposed mathematical model was accomplished using flight test data of an articulated micro air vehicle obtained from a high resolution indoor tracking facility. Analytical investigation of the gust alleviation properties of the articulated micro air vehicle model was carried out using simulations with varying crosswind gust magnitudes. Simulations show that passive articulation in micro air vehicles can increase their robustness to gusts within a range of joint compliance. It is also shown that if articulation joints are made too compliant that gust mitigation performance is degraded when compared to a rigid system.

## 1. Introduction

**Figure 1:**
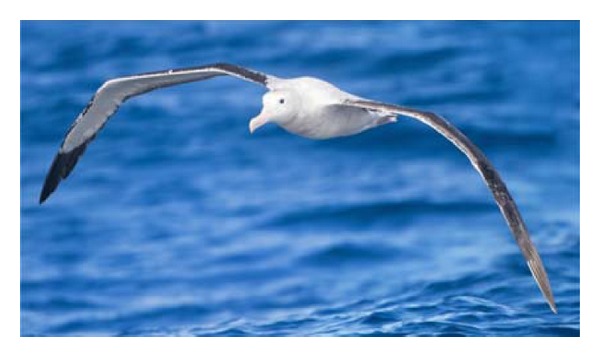
Wing roll articulation during bird flight.

Micro air vehicles (MAVs) are a class of lightweight aerial vehicles with wing spans less than 20 cm and which fly in the low Reynolds number regime. Their small size allows for several civil and military applications including remote surveillance of hazardous environments, aerial photography, and asset monitoring. However, their diminutive nature also causes many design challenges for stability, control, and robustness. Designers of unmanned aerial systems have often looked to nature for potential solutions to existing challenges such as bat-inspired algorithms for path planning [1] and wing optimization [2]. Likewise, MAV designers to improve flight dynamics and robustness have also looked to nature. An example is how turning and gliding birds allow their wing shape to respond to changes in the aerodynamic loading experienced as shown in Figure 1.

A commonly proposed advantage of passive wing changes is its ability to alleviate disturbances to wind gusts. This potential feature is highly attractive to MAV designers as vehicle size and weight continue to decrease. Micro air vehicles with passive wing deflection through material flexibility have been studied over the past two decades by several researchers [3–7] due to their perceived advantages in agility and size. This earlier work on articulated MAVs was concerned with exploring the various types and configurations that such a flier can take [3] and the issue of whether such platforms are indeed feasible and controllable [4].

The promising results of wing flexibility have spurred a new type of MAV called the articulated MAV. An articulated MAV system is comprised of a rigid MAV frame with the main wing physically divided into spanwise segments joined together by joints [8–12]. Wing articulation has the specific advantage of providing the MAV designer with a high level of control of bending stiffness and location. Work done in the area of articulated MAVs has been largely based on the vast background knowledge available for rigid MAVs. Additional investigations of the several advantages wing articulation provides the MAV include MAV turning performance [13], steady and dynamic stability as well as flight control [14], vertical/updraft wind gust alleviation [8], shape optimization by actively moving the wings to new positions [10], and mission optimization using optimal MAV configurations [11]. Researchers such as Abdulrahim and Lind [15] studied changes that occur to the trim conditions as the MAV wing deflections change in wing loading and due to the control effort. They used a shoulder-elbow concept implemented in a variable gull-wing aircraft and provided a generalized modeling framework and control synthesis for enabling autonomous operation. Recently, work done by Webb and Costello [8] used assumed wing coefficients and a vortex lattice method code to provide the changing fluid forces acting on the MAV to analytical simulations that investigated the flight dynamics of an articulated wing MAV with wings hinged at the fuselage root. Using an 18 degree-of-freedom (DOF) articulated MAV model formulated by a joint constraint enforcing controller, they reported the ability of the articulated design to provide reduced gust sensitivity during flight to an upward wind gust acting along the right wing. They also reported smaller lateral deviation in the articulated MAV when compared to the rigid MAV.

Analysis of articulated MAVs begins with an appropriate multi-DOF dynamic model. Dietl and Garcia [16] presented a purely analytical coupled vehicle dynamics/aerodynamics model for longitudinal flight in an ornithopter to analyze flight dynamics patterns for predetermined wing kinematics. As an alternative, Leylek et al. [17] used automatic generation of a multibody air vehicle simulation where 6-DOF bodies formed the basic kernels. The kernels were “glued” together using joint connection constraint forces and moments computed with a nonlinear controller that guarantees global stabilization of all constraints. In this paper an efficient analytic 8-DOF articulated MAV model is proposed which has computational benefits when compared to [17]. Importantly, the proposed model is compared to an experimental articulated MAV using a high resolution indoor motion tracking facility similar to studies [18–23]. Through comparison of the proposed model and experimental MAV, the model is validated and model coefficients are identified. Finally, the validated articulated MAV model is used to explore how joint articulation design affects the overall gust mitigation performance. It is shown that a joint stiffness exists for optimal gust mitigation using passive articulation. Importantly, it is also shown that once the joint becomes too compliant, the articulation results in a degradation of the gust mitigation capability when compared to a rigid MAV.

## 2. Articulated MAV Model

**Figure 2:**
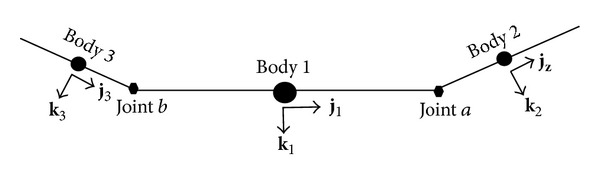
Schematic of the articulated MAV model.

The articulated MAV consists of 3 bodies acting together to form the complete MAV system as shown in Figure 2 with the center, right, and left bodies referred to as bodies 1, 2, and 3, respectively. The center body is modeled as a 6-DOF rigid body while the attached outer wings provide extra 2 DOF from their allowable rolling motion. Joints *a* and *b* in the articulated MAV are hinge joints, allowing relative roll movements between the outer bodies and the center body during flight. The deflections of the outer bodies are caused by the aerodynamic and joint forces and moments that act on the articulated bodies during flight.

### 2.1. Coordinate Frames

The central body states are its position (*x*_1_, *y*_1_, *z*_1_) and Euler roll, pitch, and yaw, (*ϕ*_1_, *θ*_1_, *ψ*_1_). The central body frame (**1**) is fixed at the mass center with its final orientation reached by following the conventional aerospace sequence of three body-fixed rotations using the Euler yaw *ψ*_1_, pitch *θ*_1_, and roll *ϕ*_1_ axis starting from the inertial frame (**I**). This transformation from the inertial to (**1**) frame can be written as

(1) $$\begin{array}{c} T_{I1}=\left[\begin{array}{ccc} c_{\theta_{1}}c_{\psi_{1}} & c_{\theta_{1}}s_{\psi_{1}} & -s_{\theta_{1}}\\ s_{\phi_{1}}s_{\theta_{1}}c_{\psi_{1}}-c_{\phi_{1}}s_{\psi_{1}} & s_{\phi_{1}}s_{\theta_{1}}s_{\psi_{1}}+c_{\phi_{1}}c_{\psi_{1}} & s_{\phi_{1}}c_{\theta_{1}}\\ c_{\phi_{1}}s_{\theta_{1}}c_{\psi_{1}}+s_{\phi_{1}}s_{\psi_{1}} & c_{\phi_{1}}s_{\theta_{1}}s_{\psi_{1}}-s_{\phi_{1}}c_{\psi_{1}} & c_{\phi_{1}}c_{\theta_{1}} \end{array}\right], \end{array}$$

using the common shorthand notation for trigonometric functions, sin(*α*) = *s*_*α*_, cos(*α*) = *c*_*α*_, and tan(*α*) = *t*_*α*_. The right and left outer wing body orientations are obtained by a single body-fixed rotation about the *i*-axis by the angles *ϕ*_21_ and *ϕ*_31_, respectively. Transformations from the right body frame (**2**) and left body frame (**3**) to the center body frame (**1**) are

(2) $$\begin{array}{c} T_{21}=\left[\begin{array}{ccc} 1\; & 0 & 0\\ 0 & c\phi_{21} & s\phi_{21}\\ 0 & -s\phi_{21} & c\phi_{21} \end{array}\right],\;\;T_{31}=\left[\begin{array}{ccc} 1\; & 0 & 0\\ 0 & c\phi_{31} & s\phi_{31}\\ 0 & -s\phi_{31} & c\phi_{31} \end{array}\right]. \end{array}$$

### 2.2. Articulated MAV Kinematics

The central body velocity and the angular velocity with respect to the inertial frame (**I**) are defined in the body frame (**1**) as

(3) $$\begin{array}{c} V_{1}=u_{1}i_{1}+v_{1}j_{1}+w_{1}k_{1}\\ \omega_{1/I}=p_{1}i_{1}+q_{1}j_{1}+r_{1}k_{1}. \end{array}$$

The translation and rotational kinematic equations for body 1 are expressed as

(4) $$\begin{array}{c} \left[\begin{array}{c} {\dot{x}}_{1}\\ {\dot{y}}_{1}\\ {\dot{z}}_{1} \end{array}\right]={T_{I1}}^{T}\left[\begin{array}{c} u_{1}\\ v_{1}\\ w_{1} \end{array}\right],\\ \left[\begin{array}{c} {\dot{\phi }}_{1}\\ {\dot{\theta }}_{1}\\ {\dot{\psi }}_{1} \end{array}\right]=\left[\begin{array}{ccc} 1 & s_{\theta_{1}}t_{\theta_{1}} & c_{\theta_{1}}t_{\theta_{1}}\\ 0 & c_{\phi_{1}} & -s_{\phi_{1}}\\ 0 & \frac{{s_{\phi }}_{1}}{c_{\theta_{1}}} & \frac{{c_{\phi }}_{1}}{c_{\theta_{1}}} \end{array}\right]\left[\begin{array}{c} p_{1}\\ q_{1}\\ r_{1} \end{array}\right]. \end{array}$$

Relative motion between the bodies in the articulated system is related to the hinge movements at the joints. The angular velocity of body 2 is defined as

(5) $$\begin{array}{c} \omega_{2}=p_{2}i_{2}+q_{2}j_{2}+r_{2}k_{2}. \end{array}$$

The relative angular velocity of body 2 with respect to body 1 is then

(6) $$\begin{array}{c} \omega_{2/1}={\dot{\phi }}_{21}i_{2}=\left(p_{2}-p_{1}\right)i_{2}. \end{array}$$

The angular velocity of the right body can be redefined in terms of the center body's angular velocity and the allowable joint constraint dynamics as shown below:

(7) $$\begin{array}{c} \omega_{2}=\;T_{12}\omega_{1/I}+\omega_{2/1}. \end{array}$$

Substituting (2) and (6) into (7) gives the angular velocity of body 2 as

(8) $$\begin{array}{c} \omega_{2}=\;\left[\begin{array}{c} p_{2}\\ q_{2}\\ r_{2} \end{array}\right]=\left[\begin{array}{c} p_{2}\\ c\phi_{21}q_{1}+s\phi_{21}r_{1}\\ -s\phi_{21}q_{1}+c\phi_{21}r_{1} \end{array}\right]. \end{array}$$

Finally, differentiating (8) with respect to the inertial frame provides the angular acceleration for body 2:

(9) $$\begin{array}{c} \alpha_{2}=\left[\begin{array}{c} {\dot{p}}_{2}\\ {\dot{q}}_{2}\\ {\dot{r}}_{2} \end{array}\right]=\left[\begin{array}{c} {\dot{p}}_{2}\\ {\hat{T}}_{12}\alpha_{1}+{\dot{\phi }}_{21}{\hat{\hat{T}}}_{12}\omega_{1} \end{array}\right], \end{array}$$

where

(10) $$\begin{array}{c} {\hat{T}}_{12}=\left[\begin{array}{c} 0\\ 0 \end{array}\begin{array}{cc} \;\;c\phi_{21} & s\phi_{21}\\ \;\;-s\phi_{21} & c\phi_{21} \end{array}\right],\\ {\hat{\hat{T}}}_{12}=\left[\begin{array}{c} 0\\ 0 \end{array}\begin{array}{cc} \;\;-s\phi_{21} & c\phi_{21}\\ \;\;-c\phi_{21} & -s\phi_{21} \end{array}\right]. \end{array}$$

Equations (5)–(10) can be similarly formed for body 3 by replacing the subscript.

### 2.3. Articulated MAV Dynamics

**Figure 3:**
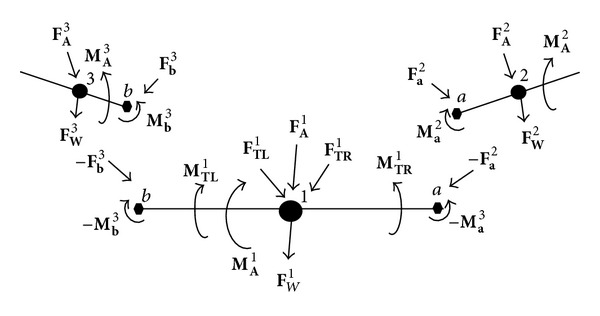
Free body diagram schematic showing the weight and aerodynamic and joint forces and moments acting on the articulated MAV.

The translational equations of motion are formed by equating the time derivative of the linear momentum with the total forces acting on each body while rotational equations of motion are formed by equating the time derivative of the angular momentum with the total moments for each body. Forces and moments acting in the system include weight **F**_**W**_^**i**^, aerodynamic forces and moments **F**_**A**_^**i**^, **M**_**A**_^**i**^, joint forces and moments **F**_**a**_^**i**^, **M**_**a**_^**i**^, **F**_**b**_^**i**^, **M**_**b**_^**i**^, and thrust and moments from right and left propellers, **F**_**TR**_^**i**^, **F**_**TL**_^**i**^, **M**_**TR**_^**i**^, **M**_**TL**_^**i**^, where the superscript *i* = 1,2, 3 represents the body and frame the vector is expressed in.  Figure 3 shows a schematic of forces and moments for the articulated MAV.

Formation of translation and rotation dynamics for each body results in the following six equations:

(11) $$\begin{array}{c} F_{A}^{1}+F_{TR}^{1}+F_{TL}^{1}+F_{W}^{1}-{T_{21}F}_{a}^{2}-{T_{31}F}_{b}^{3}=m_{1}a_{1/I}, \end{array}$$

(12) MA1+MTR1+MTL1+MW1+SR1A1FA1−T21Ma2  −T31Mb3=ddtH1/I,

(13) $$\begin{array}{c} F_{A}^{2}+F_{W}^{2}+F_{a}^{2}=m_{2}a_{2/I}, \end{array}$$

(14) $$\begin{array}{c} M_{A}^{2}+M_{W}^{2}+S_{R_{2A}}^{2}F_{A}^{2}+M_{a}^{2}=\frac{\text{d}}{\text{d}t}H_{2/I}^{}, \end{array}$$

(15) $$\begin{array}{c} F_{A}^{3}+F_{W}^{3}+F_{b}^{3}=m_{3}a_{3/I}, \end{array}$$

(16) $$\begin{array}{c} M_{A}^{3}+M_{W}^{3}+S_{R_{3A}}^{3}F_{A}^{3}+M_{b}^{3}=\frac{\text{d}}{\text{d}t}H_{3/I}^{}, \end{array}$$

where **a**_**i**/**I**_ is the acceleration of the *i*th body and **H**_**i**/**I**_ is the angular momentum of the *i*th body with respect to the inertial frame. It is noted that vector cross products are represented using the product of a skew-symmetric matrix and a column vector such that for two vectors **A** and **B** expressed as $A_{\;}={\left[\begin{array}{ccc} A_{x} & A_{y} & A_{z} \end{array}\right]}^{T}$ and $B_{\;}={\left[\begin{array}{ccc} B_{x} & B_{y} & B_{z} \end{array}\right]}^{T}$ both expressed in the **j** reference frame, **A** × **B** is written as

(17) $$\begin{array}{c} S_{A}^{j}B=\left[\begin{array}{ccc} 0 & {-A}_{z} & A_{y}\\ A_{z} & 0 & -A_{x}\\ -A_{y} & A_{x} & 0 \end{array}\right]\left[\begin{array}{c} B_{x}\\ B_{y}\\ B_{z} \end{array}\right]. \end{array}$$

In addition, the nomenclature for a distance vector from *j* to *k* is written as **R**_**jk**_; that is, **R**_2**a**_ is the distance vector from the mass center of body 2 to joint *a*.

In order to formulate the multibody equations of motion, accelerations bodies 2 and 3 need to be expressed in terms of the central body accelerations. The mass center acceleration of body 2 in terms of body 1 is

(18) a2/I=a1/I  +Sω1V1+SRa11α1−Sω1Sω1Ra1−SRa22α2+Sω2Sω2Ra2c,

where

(19) $$\begin{array}{c} S_{\omega }^{i}=\left[\begin{array}{ccc} 0 & -r_{i} & q_{i}\\ r_{i} & 0 & -p_{i}\\ -q_{i} & p_{i} & 0 \end{array}\right],\;i=1,2,3. \end{array}$$

Using the angular acceleration for body 2 in (9) gives

(20) $$\begin{array}{c} S_{R_{a2}}^{2}\alpha_{2}={\tilde{T}}_{12}{\dot{p}}_{2}+{\tilde{\tilde{T}}}_{12}{\hat{T}}_{12}\alpha_{1}+{\tilde{\tilde{T}}}_{12}{\dot{\phi }}_{21}{\hat{\hat{T}}}_{12}\omega_{1}, \end{array}$$

where

(21) $$\begin{array}{c} {\tilde{T}}_{12}=\left[\begin{array}{c} 0\\ R_{a2,z}\\ -R_{a2,y} \end{array}\right],\;\;{\tilde{\tilde{T}}}_{12}=\left[\begin{array}{cc} -R_{a2,z} & R_{a2,y}\\ 0 & -R_{a2,x}\\ R_{a2,x} & 0 \end{array}\right]. \end{array}$$

Expressing (13) in terms of only central body accelerations using (18) and (19) results in

(22) m2TI2a1/I+m2T12SRa11α1−m2T12Sω1Sω1Ra1+m2Sω2Sω2Ra2  −Fa2−m2T~12p˙2−m2T~~12T^12α1 =FW2+FA2+m2T~~12ϕ˙21T^^12ω1.

Likewise, (14) can be expressed in terms of body 1 angular accelerations as

(23) I~~2T^12α1+I~2  p˙2−SRa22Fa2−Ma2  =MA2−I~~2ϕ˙21T^^12ω1−Sω2I2ω2,

where to facilitate matrix multiplication, the inertia matrix of bodies is divided into the 3 × 2 and 3 × 1 submatrices:

(24) $$\begin{array}{c} I_{2}=\left[\begin{array}{ccc} I_{21} & 0 & 0\\ 0 & I_{22} & 0\\ 0 & 0 & I_{23} \end{array}\right]=\left[\begin{array}{cc} I_{21} & 0\\ 0 & I_{22}\\ 0 & 0 \end{array}|\begin{array}{c} 0\\ 0\\ I_{23} \end{array}\right]=\left[\begin{array}{cc} {\tilde{I}}_{2} & {\tilde{\tilde{I}}}_{2} \end{array}\right]. \end{array}$$

Similarly, (15) and (16) can be put in the form of (22) and (23) by repeating ((18)–(21), (24)) using body 3 and joint *b* rather than body 2 and joint *a*.

### 2.4. Joint Moments

Since joints *a* and *b* are constrained in the pitch and yaw axes, we can split the internal joint moment acting at the joints into a known component *M*_*ax*_ and the two unknown constraint components *M*_*ay*_, *M*_*az*_. Modeling the known joint resistance as proportional to the relative roll and roll rates between the central body, the joint momenta at *a* and *b* can be written in matrix form as

(25) Ma2=I~Max+I~~Mayz2=−I~[Caϕ˙21−Ka(ϕ21−ϕ210)]+I~~Mayz2Mb3=I~Mbx+I~~Mbyz3=−I~[Cbϕ˙31−Kb(ϕ31−ϕ310)]+I~~Mbyz3,

where

(26) $$\begin{array}{c} \tilde{I}=\left[\begin{array}{c} 1\\ 0\\ 0 \end{array}\right],\;\;\;\tilde{\tilde{I}}=\left[\begin{array}{cc} 0 & 0\\ 1 & 0\\ 0 & 1 \end{array}\right], \end{array}$$

*ϕ*_21_0__ and *ϕ*_31_0__ are the trim roll angles of bodies 2 and 3, $M_{ayz}^{2}={\left[\begin{array}{cc} M_{ay} & M_{az} \end{array}\right]}^{T}$, and  $M_{byz}^{3}={\left[\begin{array}{cc} M_{by} & M_{bz} \end{array}\right]}^{T}$.

### 2.5. Final Equations of Motion

Formation of the final nonlinear dynamic equations of motions for the 8-DOF articulated MAV is achieved by isolating the unknown state derivatives (**a**_1/**I**_, **α**_1_, ${\dot{p}}_{2}$, ${\dot{p}}_{3}$) and unknown constraints (**F**_**a**_, **F**_**b**_, **M**_**ayz**_, **M**_**byz**_) from the known components in (11) and (12), (22) and (23), and the equivalent equations for body 3. The six equations can be written in the matrix form $A\dot{x}\;=\;B$ where

(27) x˙=[a1/Iα1p˙2p˙3Fa2Fb3Mayz2Mbyz3]T,B=[B1B2B3B4B5B6]T,A=[m1TI1O3×3O3×1O3×1T21T31O3×2O3×2O3×3I1O3×1O3×1SRa11T21SRb11T31T21I~~T31I~~m2TI2−m2T~~I2T^12+m2TI2SRa11−m2T~I2O3×1−E3×3O3×3O3×2O3×2O3X3I~~2T^12I~2O3×1−SRa22O3×3−I~~O3×2m3TI3−m3T~~i3T^13+m3TI3SRb11O3×1−m3T~I3O3×3−E3×3O3×2O3×2O3×3I~~3T^13O3×1I~3O3×3−SRb33O3×2−I~~],B1=FW1−m1Sω1V1+FA1+FTR1+FTL1,B2=MA1−Sω1I1ω1+MTR1+MTL1−T21I~Max2−T31I~Mbx3,B3=FW2+T21FA2+m2T12Sω1Sω1Ra1+m2ϕ˙21T~~12T^^12ω1−m2T21Sω2Sω2Ra2−m2Sω1V1,B4=MA2−Sω2I2ω2−ϕ˙21I~~2T^^12ω1+I~Max2,B5=FW3+T31FA3+m3T13Sω1Sω1Rb1+m3ϕ˙31T~~13T^^13ω1−m3T31Sω3Sω3Rb3−m3Sω1V1,B6=MA3−Sω3I3ω3−ϕ˙31I~~3T^^13ω1+I~Max3,

with **O**_**m**×**n**_ representing a zero matrix with *m* rows and *n* columns and **E**_3×3_ representing a 3 × 3 identity matrix. The six block rows in the system $A\dot{x}=B$ correspond to the force and moment equations for the three bodies making up the articulated system. Row 1 is forces acting on the mass center of the central body 1 in the (**1**) frame; rows 2 is moments acting about central body 1 in the (**1**) frame. Similarly, row 3 and 4 are the forces and moments acting on body 2 while rows 5 and 6 are the forces and moments acting on body 3. The 8-DOF equations of motion for the articulated MAV can be determined by solving the above dynamic equations in combination with the kinematic equations in (4) and (6).

## 3. Model Estimation

### 3.1. Experimental Articulated MAV

**Figure 4:**
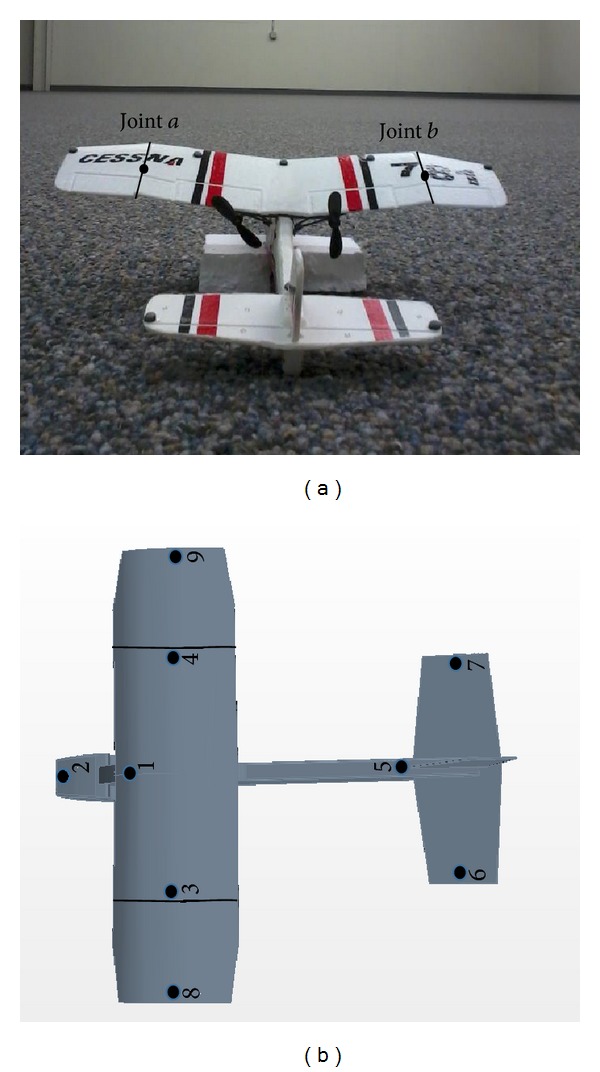
Rigid and articulated MAV models showing marker positions.

The MAV that was flight tested is a 9-gram, dual propelled platform, 18.5 cm in length and 20 cm in wingspan. The actual articulated MAV and the reflective marker placements are shown in Figure 4 with physical properties provided in Table 1.

The 8-DOF MAV configuration is achieved by splitting the main wing into three separate sections and reattaching them back together along the middle of the cut plane with a plastic strip that acts as the joint spring mechanism constraining the relative motion between the wings to a rolling motion. The MAV is controlled by two propellers with their thrust vector direction aligned with the positive *x*-axis of the center body frame. The two propellers rotate in opposite directions to cancel out most of the thrust-induced torque.

### 3.2. Motion Capture Facility

**Figure 5:**
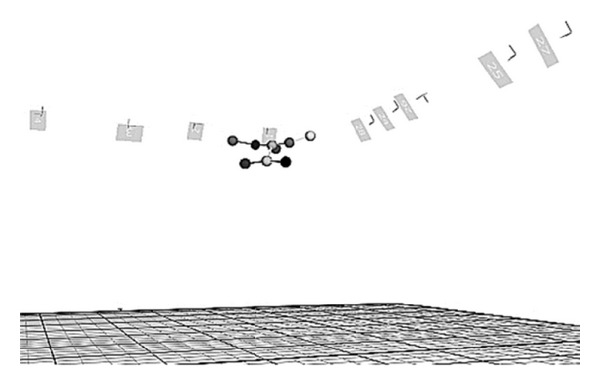
Articulated MAV flight test in the VICON environment showing individual reflective marker positions.

The UAHuntsville Autonomous Tracking and Optical Measurement (ATOM) lab is a powerful digital tracking solution that provides very high data accuracy for 3D applications. The ATOM lab has a 16 m ×10 m ×4 m unobstructed capture volume for tests and achieves accurate motion capture using 33 VICON T40 series IR cameras. The cameras use infrared LED illumination to achieve marker location tracking of nine markers on the articulated MAV. The large numbers of high resolution cameras allow 1.5 mm tracking accuracy over the entire capture volume. A flight test image of the articulated MAV in the VICON environment along with the individual reflective markers is shown below (Figure 5).

Instantaneous positions of markers on the MAV are logged at a frequency of 100 Hz. The dataset is then postprocessed to give the eight state and state derivatives for the articulated MAV during any given flight test.

### 3.3. System Identification Using the Output Error Method

The output error method is a widely used time-domain estimation method for aircraft parameters estimation from flight test data [24] and is used here to estimate the articulated MAV aerodynamic coefficients. Estimates for the unknown parameters are used with the system model to predict trajectories. A residual error is computed using the measured and predicted trajectories. Using a Newton-Raphson method, updates to the unknown parameters which reduce the residual error are found. This process leads to a nonlinear optimization problem in which an optimal set of parameters are chosen to describe the nonlinear model of the system in question.

**Figure 6:**
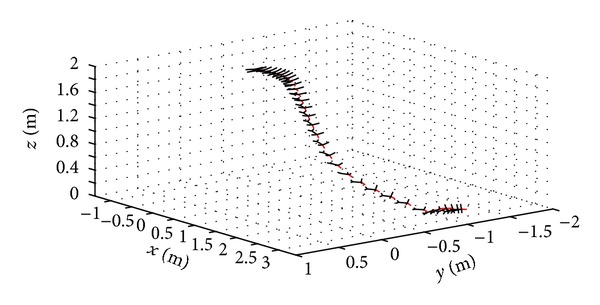
Flight test results for articulated MAV during gliding flight.

Experimental gliding data of the articulated MAV described in Section 3.1 was collected by hand launching the vehicle and allowing it to glide to the ground. The collected trajectory and orientation data are shown in Figure 6 where the MAV position and orientation are represented at discrete times by the images. From Figure 6 it can be seen that after an initial increase in altitude the MAV banks and yaws while descending.

The collected trajectory data and the aerodynamic model in (28) were used within the output error method detailed in [24] to estimate the 18 aerodynamic coefficients used to model the articulated MAV. The aerodynamic model was applied individually to each of the three lifting surface, and used to calculate aerodynamic forces and moments **F**_**A**_^**i**^, **M**_**A**_^**i**^, for *i* = 1 to 3 that appear in Figure 3 and the final MAV equations. Since each section had identical cross sections the 18 aerodynamic parameters remained the same for each surface:

(28) $$\begin{array}{c} C_{L}=C_{L0}+C_{L\alpha }\alpha +C_{Lq}\frac{q\overset{-}{c}}{2V},\\ C_{Y}=C_{y\beta }\beta +C_{yp}\frac{pb}{2V}+C_{yr}\frac{rb}{2V},\\ C_{D}=C_{D0}+C_{D\alpha }\alpha +C_{Dq}\frac{q\overset{-}{c}}{2V},\\ C_{l}=C_{l\beta }\beta +C_{lp}\frac{pb}{2V}+C_{lr}\frac{rb}{2V},\\ C_{m}=C_{m0}+C_{m\alpha }\alpha +C_{mq}\frac{q\overset{-}{c}}{2V},\\ C_{n}=C_{n\beta }\beta +C_{np}\frac{pb}{2V}+C_{nr}\frac{rb}{2V}. \end{array}$$

**Figure 7:**
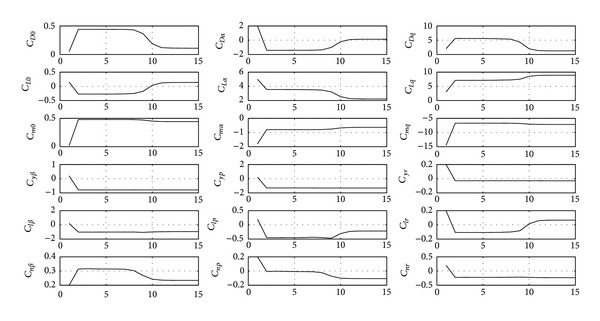
Convergence of maximum likelihood model parameter estimates.

Convergence histories of the maximum likelihood parameter estimates from the output error method are shown below with convergence reached within 15 iterations (Figure 7). The final aerodynamic parameters are provided in Table 2.

**Figure 8:**
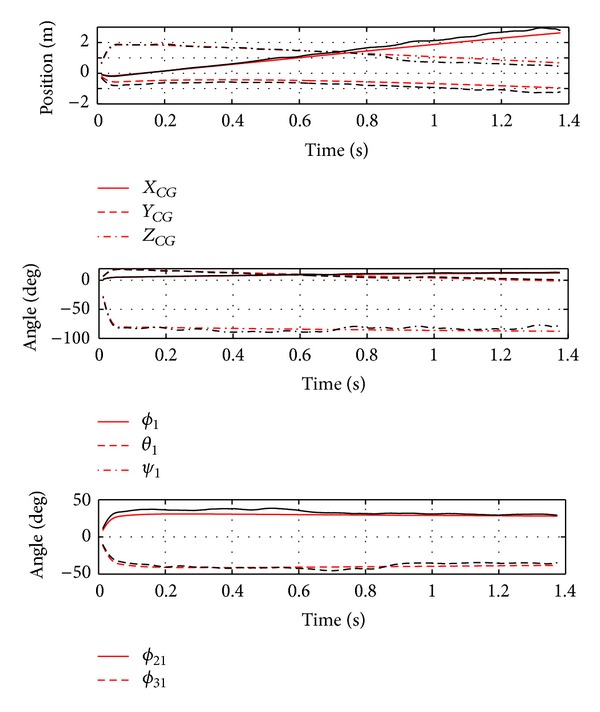
Flight test and model simulation comparison for gliding articulated MAV.

Figure 8 provides a comparison of the experimental data with the 8-DOF articulated MAV model's prediction of mass center positions, central body orientation, and outer body flapping angles. The model provides a satisfactory match with the gliding flight test for the test duration and captures the qualitative nature of the flight. In addition to reconstructing the trajectory, the flapping dynamics are well represented. This feature can be used to help analyze the detailed dynamics that occur during the response to wind gusts and understand how articulation can be designed to improve overall performance.

## 4. Articulation MAV Dynamics

**Figure 9:**
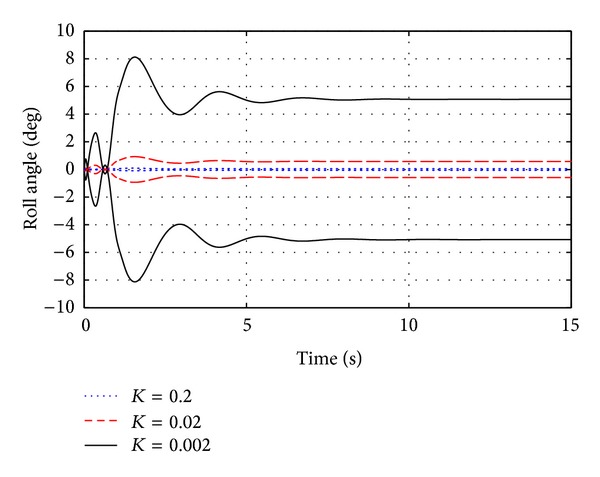
Outer roll angles for a fixed damping ratio and varying spring stiffness.

A significant difference between the rigid and articulated MAV during flight is the changes that occur in outer body roll angles. The rigid MAV has fixed roll angles as defined by its configuration whereas the articulated MAV will have nonzero outer roll angles that depend on the joint spring stiffness, shape of the outer wing bodies, and the prevailing flight speed. Below is a comparison of transition to a steady glide for different joint rotational stiffness values while holding the damping ratio constant at 0.6. As expected, the amplitude is sensitive to the joint stiffness while the response shape remains similar. From Figure 9 it is shown that by choosing the joint stiffness to be very large the articulated MAV behaves as a rigid MAV, while articulation angles can become large for sufficiently compliant joints. From a flight dynamics and design perspective the important question becomes “how compliant should the articulation joint be made?”

**Figure 10:**
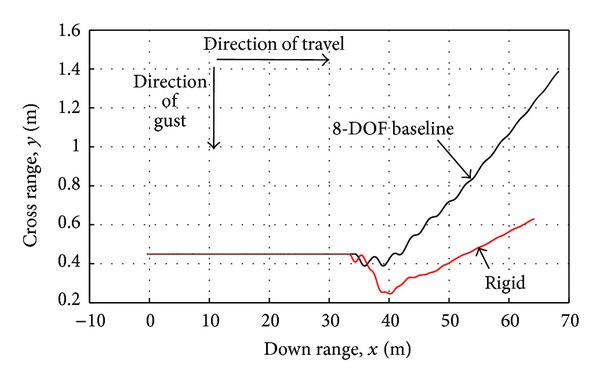
Rigid and articulated MAV trajectory in response to a 2 m/s crosswind gust.

**Figure 11:**
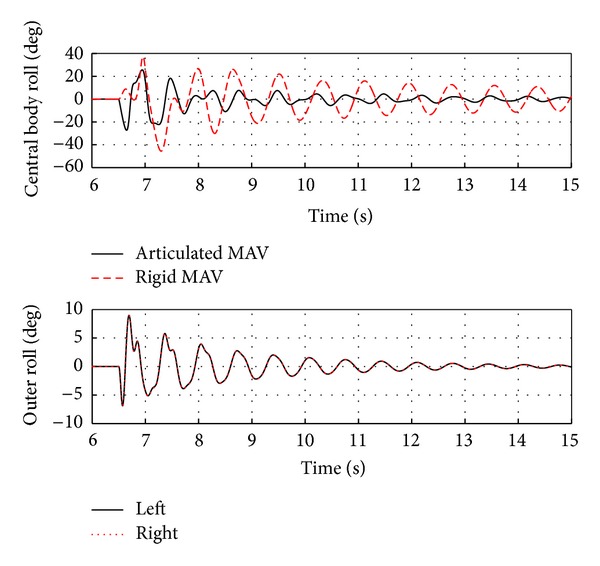
Rigid and articulated MAV center and outer body roll in response to a 2 m/s crosswind gust.

The rigid MAV and the baseline articulated MAV having a spring stiffness of 0.0216 N-m/rad and a damping coefficient of 0.0028 N-m-s/rad encountering the same 1.5 second crosswind gust of 2 m/s are compared in Figures 10 and 11.  Figure 10 illustrates two different features of the MAV response to a gust: a lateral shift from increased drag and weathercocking due to the MAVs directional stability. In response to the gust, the rigid MAV experiences 0.2 m of lateral shift while the articulated MAV has only a negligible amount. Rather than experiencing lateral shift, the articulate MAV responds to the gust mainly by turning into the crosswind.

Differences in the response can be understood by comparing the roll angles changes that occur for the two system's central bodies as shown in Figure 11. The initial roll response of the rigid MAV is positive and reaches approximately 40 degrees. In contrast, the motion of the articulated MAVs outer bodies absorbs much of the effect of the gust, resulting in almost half the roll angle for the central body. Another significant difference is that the articulated MAV central body initially rolls in the opposite direction.

**Figure 12:**
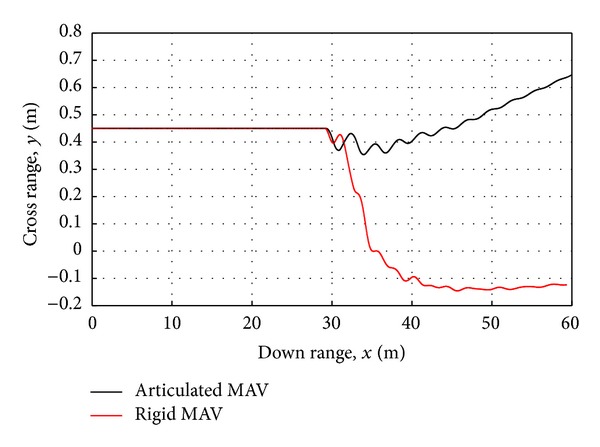
Rigid and articulated MAV trajectory in response to a 3 m/s crosswind gust.

The nature of response for both the rigid and the articulated MAV to crosswinds gust varies with gust magnitudes. For small gust amplitudes, the cross range deviations are nearly identical. As the crosswind gust magnitudes continue to increase, the MAV responds with larger deviations in its trajectory path and roll angle (Figure 10). The articulated MAV is seen to mitigate the initial crosswind gust by experiencing less central body roll and lateral shift. Increases in the gust magnitude further demonstrate the benefits of articulation as shown in Figure 12 for a 3 m/s gust of 1.5 seconds. In the case of 3 m/s, the rigid MAV experiences nearly 1 m of lateral shift while the articulated MAV experiences only 0.1 m.

**Figure 13:**
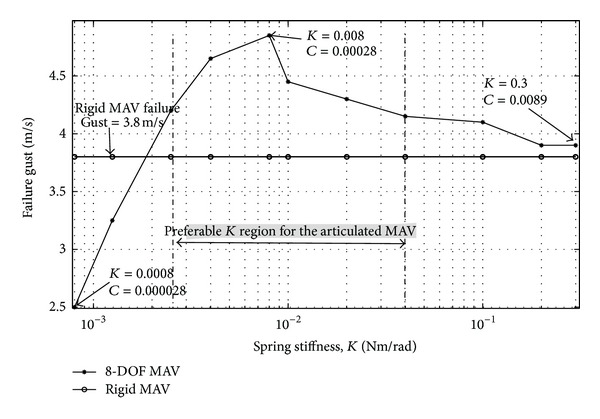
Maximum allowable crosswind gust versus joint spring stiffness plot.

While reduction of central body motion and lateral motion deviation is important for MAVs, another crucial design feature is the ability to reduce the occurrence of catastrophic failure. Failure in this context is when the MAV, due to a large amplitude crosswind, rolls past a natural recovery point of 90 degrees. The rigid MAV described here experiences catastrophic failure which occurs at a gust magnitude of 3.9 m/s.  Figure 13 compares the maximum allowable crosswind gust that is survivable for the 8-DOF articulated MAV against the rigid baseline while holding the joint damping ratio constant. The right side of Figure 13 corresponds to joint stiffness values where the articulated MAV response begins to converge to the rigid response. Significantly, it is observed that initially as the spring stiffness is relaxed to the point where articulation becomes significant, the ability of the flexibility to mitigate gust disturbances increases and larger winds can be compensated for before catastrophic failure. The maximum survivable crosswind gust that the articulated MAV can survive is 4.9 m/s, 25% higher than the rigid MAV. However, Figure 13 also shows that once the springs become too soft the articulation results in a degradation of the gust mitigation capability. As a consequence, there is a region of optimal spring stiffness values for obtaining the best passive gust response when using an articulated MAV. Designers of articulated MAVs must then carefully consider the joint compliance because as seen in Figure 13 a properly designed articulated MAV will outperform a rigid MAV; however, if the articulation joint is too compliant, the articulated MAV may not yield any improved gust mitigation.

## 5. Conclusion

An efficient 8-DOF articulated MAV model was presented and experimentally validated using motion capture data for gliding trajectories. The model was then used to analyze the gust mitigation performance of an articulated MAV compared to a rigid version. Analysis showed that the MAV trajectory response to a crosswind gust includes a lateral shift in the trajectory due to the increase in drag as the MAV rolled sideways and a weathercocking of the MAV that eventually results in a new direction of travel. As the gust magnitude increased it was shown that the articulated MAV was able to mitigate central body roll deviations which resulted in substantially less lateral trajectory deviation. In addition, it was shown that the articulated MAV was able to withstand 25% larger wind gusts before experiencing a catastrophic failure when the articulation joint compliance was designed optimally. Furthermore, analysis showed that gust mitigation of the articulated MAV initially increased as joint compliance increased; however, making the joint too compliant degraded performance when compared to the rigid MAV.

## Figures and Tables

**Table 1:** Physical properties of the articulated MAV.

Parameter	Left wing	Center body	Right wing	Units
*m*	0.00027	0.0084	0.00027	kg
*b*	0.036	0.1278	0.036	m
$\overset{-}{c}$	0.055	0.062	0.055	m
*S*	0.002	0.0079	0.002	m^2^
*I*_*xx*_	2.925*e* − 8	3.210*e* − 5	2.925*e* − 8	Kg·m^2^
*I*_*yy*_	6.815*e* − 8	7.000*e* − 5	6.815*e* − 8	Kg·m^2^
*I*_*zz*_	9.722*e* − 8	9.730*e* − 5	9.722*e* − 8	Kg·m^2^
*I*_*xz*_	0	−4.800*e* − 6	0	Kg·m^2^
*I*_*xy*_	0	−1.590*e* − 5	0	Kg·m^2^
*I*_*yz*_	0	−8.000*e* − 7	0	Kg·m^2^

**Table 2:** Estimated MAV aerodynamic and thrust coefficients.

Parameters	Value	Parameter	Value
*C*_*D*0_	0.11	*C*_*yb*_	−0.96
*C*_*Dα*_	0.14	*C*_*yp*_	−0.22
*C*_*Dq*_	1.25	*C*_*yr*_	0.07
*C*_*L*0_	0.14	*C*_*lb*_	−0.79
*C*_*Lα*_	2.22	*C*_*lp*_	−1.32
*C*_*Lq*_	8.90	*C*_*lr*_	−0.03
*C*_*m*0_	0.44	*C*_*nb*_	0.23
*C*_*mα*_	−0.64	*C*_*np*_	−0.11
*C*_*mq*_	−7.16	*C*_*nr*_	−0.23
